# Human Obesity

**Published:** 1883-10

**Authors:** 


### Human Obesity.

Last week the death was recorded of the "fattest woman in the world," a member and special curiosity of a traveling circus company in America, who appears to have been smothered in her bed by rolling upon her face, she being unable to turn upon her back without assistance. Miss Conley, the adipose prodigy in question, though probably the most enormous of her sex, weighing, as she is stated to have done, 497 lbs., fell far short of the bulk of the famous Daniel Lambert, who died in 1809, at Stamford, at the age of 40. Lambert had no less than 52 stones, 11 lbs., that is 739 lbs., or close upon half as much again as the American lady. He was 5 ft. 11 in. in height, measured 9 ft. 4 in. round his body, and 3 ft. 1 in. round his leg. It is said that he never drank any alcoholic beverage, and that he usually slept less than eight hours a day.—*British Medical Journal.*

IDOFORM.—Mr. Scherck, of Koenigsdorff, finds that one grain of carbolic acid mixed with three drachms of iodoform and one or two drops of oil of peppermint, completely disguises the iodoform smell.

